# Making Sense from Structure: What the Immune System Sees in Viral RNA

**DOI:** 10.3390/v18010128

**Published:** 2026-01-20

**Authors:** Benjamin J. Cryer, Margaret J. Lange

**Affiliations:** 1 Department of Molecular Microbiology & Immunology, School of Medicine, University of Missouri, Columbia, MO 65212, USA; bjcqxf@missouri.edu; 2 Department of Biochemistry, School of Medicine, University of Missouri, Columbia, MO 65212, USA

**Keywords:** innate immune, RNA structure, virus, pattern recognition receptor

## Abstract

Viral RNA structure plays a critical regulatory role in viral replication, serving as a dual-purpose mechanism for encoding genetic information and controlling biological processes. However, these structural elements also serve as pathogen-associated molecular patterns (PAMPs), which are recognized by pattern recognition receptors (PRRs) of the host innate immune system. This review discusses the complex and poorly understood relationship between viral RNA structure and recognition of RNA by PRRs, specifically focusing on Toll-like receptor 3 (TLR3) and Retinoic acid-inducible gene I (RIG-I). While current interaction models rely upon data generated from use of synthetic ligands such as poly(I:C) or perfectly base-paired double-stranded RNA stems, this review highlights significant gaps in our understanding of how PRRs recognize naturally occurring viral RNAs that fold into highly complex three-dimensional structures. Furthermore, we explore how viral evolution and nucleotide variations, such as those observed in influenza viruses, can drastically alter local and distal RNA structure, potentially impacting immune detection. We conclude that moving beyond synthetic models to understand natural RNA structural dynamics is essential for elucidating the mechanisms of viral immune evasion and pathogenesis.

## 1. Introduction

Viruses are under continuous selective pressure to preserve genetic information while maintaining function and escaping immune detection [1]. Genetic information is not simply stored in the nucleotide sequence by virtue of the encoded genes and proteins; the three-dimensional structural elements formed by inter-nucleotide interactions are also critical to regulation of viral replication cycles. RNA structure plays a central and highly conserved [2,3,4] regulatory role by encoding information in both the nucleotide sequence as well as the formation of higher-order structures that control numerous processes from translation to assembly [5,6,7]. Structural elements within viral RNAs can recruit and reinitiate ribosomes [8,9], tightly control frameshifting [10,11], coordinate the switch from translation to genome replication [12,13], regulate stoichiometry of new proteins and replicated genomes [14,15], and facilitate processes required for assembly of new viral particles [13,16]. Critical to these processes is the ability of the virus to conserve RNA structure and function while working to effectively evade detection by the host immune system, which encodes numerous RNA sensors.

As opposed to the adaptive immune system, the innate immune system employs germline-encoded receptors that recognize molecular patterns conserved across broad classes of microbes. These pattern recognition receptors (PRRs) are sentinels of the innate immune system, recognizing conserved molecular signatures that culminate in the initiation of responses that serve to slow replication and alert the adaptive immune system to the presence of an invader. RNA is one such conserved molecular pattern as a fundamental molecule of life. The importance of RNA as a molecular pattern is exemplified by the number of PRRs identified to date that sense RNA associated with viral genomic material and/or replication intermediates [17,18,19]. However, the immune system must take care to recognize pathogenic RNA while ignoring essential host RNAs. To differentiate viral RNA from host RNA, RNA-sensing PRRs rely on often subtle RNA features characteristic of viral or mislocalized RNA that are rare in properly processed cellular RNA [20]. The ability to distinguish self from non-self is critical, as mistakes lead to aberrant activation or inactivation, both of which can be detrimental to human health. Conversely, knowledge and control of RNA signaling mechanisms has been utilized to engineer powerful tools for public health, such as mRNA vaccines [21,22]. Despite these monumental advancements, current evidence suggests that the puzzle is not yet solved; the molecular mechanisms governing recognition of self and non-self RNA by PRRs remain incompletely understood.

A key challenge is the intrinsic and dynamic nature of RNA. Not only does RNA display a high degree of flexibility and the ability to exist in an ensemble of folded conformations, but these conformations are also highly context dependent. This dynamic nature is often integral to RNA function; for example, riboswitches are a class of structural regulatory elements that control transcription and translation by changing conformation depending on the availability of small molecules, ions, or metabolites [23]. RNA conformation can also change upon RNA–protein interactions, and there is a growing appreciation that this is not a one-sided affair, with RNA having the “riboregulatory” capability to dictate protein function as well [24,25]. Further complexity is added when considering the impact of natural RNA modifications, of which hundreds have been identified and catalogued [26], on folding dynamics and protein interactions [27]. RNA modifications are certainly incorporated in viral replication strategies [28], though which modifications and to what extent modification occurs are highly variable and still under investigation. Finally, viral evolution frequently introduces RNA sequence variation, and even single nucleotide variations can substantially alter RNA structure by disrupting both short- and long-range RNA intramolecular interactions [29].

The complexity and breadth of RNA structural space present a distinct technical challenge, creating significant gaps in our understanding of RNA/PRR interactions. Many PRRs have been well-studied in the context of model ligands, such as the synthetic RNA polyinosinic:polycytidylic acid (poly(I:C)) or perfectly base-paired double-stranded RNA stems. These ligands are powerful and instrumental tools, especially considering the technological challenge of studying RNA structure. However, there is still much to learn about mechanisms underlying recognition of naturally occurring, virus-derived RNA, which adopts diverse and highly complex structures. In this review, we will discuss the current state of knowledge of RNA molecular patterns recognized by the innate immune system, some examples of discrepancies, and variables to consider that might impact the biophysical or biochemical interactions of RNA and PRRs. Our goal is to encourage future researchers to consider the dynamics of viral RNA sequence, structure, and modifications on RNA/PRR interactions.

## 2. Sources of RNA During Viral Infection

RNA is a well-established molecular pattern for the immune system, both in single-stranded (ssRNA) and double-stranded (dsRNA) forms. ssRNA is, of course, represented by the genomes of positive- and negative-sense ssRNA viruses, as well as mRNAs generated during viral replication of all Baltimore virus classifications [30]. dsRNA originates not only from viruses with dsRNA genomes but also from intermediates generated during replication of positive-sense ssRNA genome viruses, to some extent similar products from negative-sense ssRNA viruses, and long sense–antisense hybrids from bidirectional transcription of dsDNA viruses [31,32]. Structures adopted within the genomes of ssRNA viruses, both positive- and negative-sense, often include substantial dsRNA regions, which can further be interspersed with a variety of structural elements.

Just as proteins are linear chains of amino acids that fold sequentially into secondary and tertiary structures, RNA similarly undergoes hierarchical folding into secondary structure elements, including simple structures like loops, bulges, and helices, or more complex structures like pseudoknots and G-quadruplexes, which may all form further tertiary interactions [33]. By forming complex structures, RNAs generate new properties otherwise unattainable, including catalytic activity [34]. These structures are promoted not just by canonical Watson–Crick base pairs; the 2′ hydroxyl on the ribose can act as a hydrogen donor and acceptor and promotes flexibility through the C3-endo sugar pucker, leading to additional Hoogsteen edge and sugar edge interactions that facilitate complex networks of intramolecular interactions [35]. As mentioned previously, structural elements control RNA and subgenomic RNA prevalence through common mechanisms, including splicing and frameshifting. RNA prevalence can also be used to control immunogenicity. In an interesting example, the Dengue virus 2′s genomic RNA contains a pseudoknot in the 3′ UTR, which stalls the exoribonuclease XRN1 [36]. This stall produces a short subgenomic flavivirus RNA (sfRNA) that sequesters TRIM25 and prevents RIG-I ubiquitination and activation [37]. There are multiple copies of this element in the 3′UTR, resulting in multiple subgenomic RNAs of varying length. Further, some, but not all, of these pseudoknots are disrupted by oscillating mutations in what seem to be host-specific fitness adaptations governing arthropod or mammalian infection [38,39].

It is also important to acknowledge that not all potentially immunogenic RNA present during the course represents a perfect consensus sequence or structure, or even serves the purpose of replication. Viruses often exist as a highly variable quasispecies [40], which could result in additional structured ssRNA and dsRNA sources present during the course of an infection. Indeed, defective interfering particles often contain non-standard viral genomes such as copy-back viral genomes [41,42,43]. Viral replication can also cause mislocalization or defects in post-transcriptional processing of host nucleic acid through hijacking of host pathways, providing additional potential RNA sources for sensing [44,45,46]. The existence of these different sources of RNA during viral infection is interesting in the context of RNA sensing, calling into question exactly which RNAs are being recognized by RNA-sensing PRRs. Further complicating the idea of RNA sensing, viral RNAs are often associated with and protected by RNA-binding proteins [47,48], and many viruses encode defenses against the innate immune system by effectively shutting down sensing proteins and pathways [49,50,51]. Considering most studies do not use methods that directly identify RNA/PRR interactions, it may be inappropriate to assume the source of activation for these receptors without further high-resolution studies.

## 3. RNA-Sensing Pattern Recognition Receptors

Mammals have numerous RNA-sensing PRRs that act via a wide variety of molecular mechanisms, sense many distinct RNA patterns, and are active in multiple cellular compartments to cast a wide net against viruses. Well-studied examples in humans include the toll-like receptors (TLRs) TLR3, TLR7, and TLR8; the RIG-I-like receptors (RLRs), retinoicacid-inducible gene I (RIG-I), melanoma differentiation associated gene 5 (MDA5), and laboratory of genetics and physiology 2 (LGP2);protein kinase R (PKR); and the oligoadenylate synthases (OAS1, 2, 3, and L). Other RNA sensors are currently being further explored, such as several NLRP proteins and ZBP1 [17]. Classical DNA sensors, such as cGAS, also have been reported to have RNA-sensing potential when supported by additional RNA-binding protein cofactors [52]. Similarly, a growing number of proteins, many with previously assigned functions, are also gaining recognition for their roles in immune sensing—namely the DEAD-box helicase family [53,54]. Some members of this family seemingly enhance or otherwise regulate established PRRs. Others may directly sense RNA alone, such as DDX23 [55], or in multiprotein complexes like DDX1, DDX21, and DHX36 [56]. However, the integral role of many of these proteins in cellular homeostasis makes it difficult to resolve their exact mechanisms; there is still much to learn about their many functions, but careful study is needed.

The aim of this review is not to comprehensively review each RNA-sensing PRR, but rather to encourage consideration of the dynamic and heterogeneous nature of RNA ligands in the study of these proteins. Therefore, while numerous RNA sensors have been described and are worthy of extensive further study, our discussion here focuses primarily on two of the most well-studied RNA-sensing PRRs, TLR3 and RIG-I. For more information on the other RNA sensors, we direct reader attention to a number of excellent recently published reviews [17,19,53,57,58]. Our discussion will include our current understanding of recognition of RNA by TLR3 and RIG-I, as well as observations that do not fit within these canonical recognition models.

### 3.1. Toll-like Receptor 3 (TLR3)

**Figure 1:**
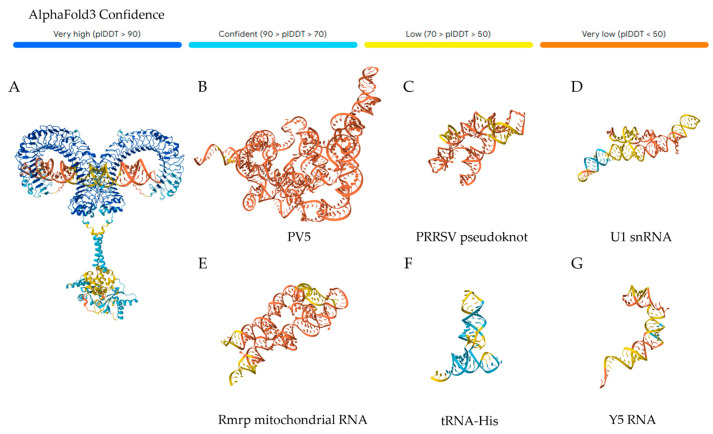
(**A**) An AlphaFold3 prediction of a canonical TLR3 dimer in complex with an arbitrary dsRNA stem loop. (**B**–**G**) AlphaFold3 predictions of the tertiary structure of the indicated RNA species reported to activate TLR3 [62,83,90,91,93]. The color indicates the predicted Local Distance Difference Test (pLDDT) score per residue.

TLR3 is an endosomal transmembrane protein widely expressed and recognized for its role in sensing dsRNA, a critical PAMP derived from sources including viral genomes [59,60,61,62,63], replication intermediates [64], endogenous RNAs [65], and RNAs released from damaged cells [66,67]. For signaling to occur, two TLR3 monomers dimerize around their dsRNA ligand in the endosomal lumen, bringing the cytosolic Toll/Interleukin 1 (TIR) signaling domains into close proximity for interaction with adaptor proteins that drive initiation of a signaling cascade [68] (Figure 1A). TLR3 binding to RNA is dependent on the lowering of endosomal pH below 6.5 due to two conserved clusters of histidine residues on each monomer that constitute the primary binding sites [69,70]. TLR3 has been demonstrated to bind dsRNA in a sequence-independent manner driven by electrostatic interactions between these histidine residues and the phosphate backbone of dsRNA [69,71]. Individual mutation of most of these histidine residues, with the exception of H108, has been shown to abolish TLR3-mediated signaling upon delivery of dsRNA stems or synthetic dsRNA in the form of poly(I:C) [71]. However, the impact of these mutations on sensing has not been explored in the context of viral infection. Crystal structures of the TLR3 ectodomain in complex with poly(I:C) illustrate how linear dsRNA binds to TLR3, corroborating early data indicating that a dsRNA stretch of >45 bp is necessary for stable TLR3 dimerization [68]. The maximum distance between dsRNA interacting residues in the TLR3 homodimer is ~110 Å, corresponding to four turns of an A-form helix [68,72]. A-form DNA is capable of blocking dsRNA binding but does not generate IFN-β [73]. This may be because TLR3 N541, another residue critical for activation, forms hydrogen bonds with an RNA 2′ hydroxyl [72,74]. Without this interaction, a stable duplex may not form. Recent models have demonstrated multimerization of TLR3 dimers along the length of extended dsRNAs [75,76,77], likely enhancing signaling by locally enriching downstream signaling effectors.

While TLR3 is well-established as a dsRNA sensor, most molecular characterizations have exclusively utilized synthetic TLR3 ligands (e.g., poly(I:C), dsRNA duplexes). There are some discrepancies even within synthetic duplexes; poly(I:C) was found early on to be much more stimulatory than poly(A:U) [73]. Additionally, the involvement of TLR3 in the immune response to ssRNA viruses, including IAV [78], SARS-CoV-2 [79], and others [59,63,80,81,82], is well-documented, bringing into question which natural RNAs are sensed during infection. Indeed, very few naturally occurring TLR3-binding RNAs have been identified [62,66,83]. Tatematsu et al. [62] reported that TLR3 can recognize highly structured RNA within the poliovirus (PV) genome, including non-canonical dsRNA regions containing bulges and internal loops. Two segments, PV5 (Figure 1B) and PV6, were shown to bind and activate TLR3. Limited analysis of five sub-segments of PV5 yielded two non-stimulatory RNAs and three stimulatory RNAs; however, binding was not evaluated for all non-stimulatory RNAs, and interaction sites on TLR3 were not identified. Porcine TLR3 has also been shown to recognize a pseudoknot structure within the 3′ UTR of Porcine Reproductive and Respiratory Syndrome Virus (PRRSV) [83] (Figure 1C), demonstrating that recognition of ssRNA structures applies beyond hTLR3. However, structured ssRNA regions of the Hepatitis C Virus (HCV) were unable to activate TLR3 in cells [64,84].

Another layer of complexity lies in the common use of recombinant mouse TLR3 ectodomain [69,74,85,86,87,88], despite significant differences between mouse and hTLR3 [74,89]. Mouse TLR3 has also been reported to bind small and structured ssRNAs, including U1 snRNA [90] (Figure 1D), Rmrp mitochondrial RNA [91] (Figure 1E), and siRNA [92], but the latter could not be replicated with human TLR3 [92], and the former examples were not tested in human contexts. Similarly, mouse B-cells were reported to sense the Y-RNA, Y5 (Figure 1F), and tRNA_His_ (Figure 1G) in a TLR3-dependent manner [93]. Another study noted that the human Y-RNA, Y3, was recognized by mouse TLR3 [94]. One explanation for discrepancies between mouse and human TLR3 may be that mouse TLR3 is more stable in a “lapped” conformation than human TLR3, where ectodomains overlap instead of stagger [91,95], which has not yet been demonstrated for hTLR3. Interestingly, human TLR3 was recently demonstrated to bind to and be activated by the snRNA U6 in cells [96]. The authors also determined that this interaction depends on the residues in Leucine-Rich Repeat (LRR) 21 (amino acids 587-608), while poly(I:C) binding remained dependent upon the canonical residues in LRR 20.

Collectively, these studies highlight gaps in our understanding of how TLR3 interacts with ssRNAs containing complex, biologically relevant structures prevalent during viral infections. Other elements of TLR3 biology that have not been fully explored may also influence the molecular interactions between TLR3 and RNA. TLR3 is heavily glycosylated on its non-ligand-binding surfaces, and glycosylation patterns vary depending on cell type [97], but the role of glycosylation in TLR3 function is still unclear. A glycan located at Asn413 may form a direct contact with the RNA ligand [87], and mutation of this residue decreases signaling. Interestingly, the common TLR3 L412F polymorphism is directly adjacent to a putative glycosylation site, which has been postulated to underlie differential regulation of infection in the presence of this SNP [98,99,100]. Lastly, TLR3 must also be cleaved by endosomal cathepsins to signal properly [101], but the functional benefit of TLR3 cleavage is not fully understood. It may provide some flexibility to the RNA–TLR3 interactions, especially in the context of structured ssRNAs, but this has not yet been experimentally confirmed.

### 3.2. Retinoic Acid-Inducible Gene I (RIG-I) and RIG-I-like Receptors

RIG-I is perhaps the most well-studied PRR in terms of biophysical and biochemical requirements for signaling. RIG-I is localized to the cytosol, where it plays a major role in generating antiviral responses upon infection by numerous viruses [102]. Like TLR3, RIG-I responds to dsRNA and is also activated by low molecular weight poly(I:C) [103]. However, RIG-I has been shown to have distinct requirements as compared to TLR3, being variably activated by RNAs with (or without) a 5′ppp, variable capped RNAs, dsRNA regions, and potentially U-rich regions [104,105,106,107,108,109,110,111,112]. The protein contains two auto-repressed caspase activation and recruitment domains (CARDs), an RNA-dependent ATPase motor domain often referred to as helicase domains, and a carboxy-terminal domain (CTD) [113,114]. Upon recognition of RNA by contacts made with the triphosphate or diphosphate, RIG-I undergoes a conformational change that releases its sequestered CARDs. These CARDs are the platform for oligomerization with other active RIG-I molecules that stabilize downstream signaling [102]. Several studies have purported that all RNA binding to RIG-I is sufficient to temporarily release the CARDs and that a conformation change that alters helicase activity is instead part of a kinetic regulatory system [115,116,117]. In support of this idea, several RIG-I SNPs affect the stability of CARD sequestration to different degrees, indicating that the length of time that a CARD is released is a critical factor in signaling [118,119]. There may also be a role for reaching the opposite terminus of the RNA in a timely manner, contributing to length-dependent sensing [120], which would correspond to the observations that RIG-I prefers to sense shorter transcripts. Notably, RIG-I forms a tunnel-like structure around the end of the dsRNA duplex, leaving space at the far end of the tunnel for a larger diversity of RNA structures [121].

Several studies have sought to identify natural ligands for RIG-I, primarily utilizing PAR-CLIP to identify viral genomic regions that bind RIG-I during infection [106,107,122]. Subgenomic defective interfering RNAs were shown to be preferentially associated with RIG-I as compared to full-length genomes. Indeed, defective genome particles tend to form short panhandle motifs that can activate RIG-I [123]. These have been observed in many viral species, including Sendai virus [107] and influenza A [124,125]. Some full-length genome segments associated with RIG-I, including interior genomic regions downstream of the 5′ppp, indicating that RNAs other than blunt-end dsRNAs are capable of binding and activating RIG-I [126]. RIG-I has also been reported to initiate responses following stimulation by circular RNAs lacking a 5′ end, although this is controversial [127] and may depend on associated RNA-binding proteins [128] or naturally occurring RNA modifications [129]. Recently, RIG-I has been reported to be activated by a G-quadruplex structure found in an endogenous RNA. The authors posit that this activity may be due to a quadruplex-dependent GTP-binding pocket that provides GTP as the activating triphosphate source [130]. The influenza A virus panhandle also contains a unique kink, inducing a sharp helical bend, which has been observed to activate RIG-I without a need for a 5′ triphosphate [131]. Specific U-rich regions of the hepatitis C virus preferentially activate RIG-I and maintain activity when transferred to other sequences [132,133], and similar activation was observed for AU-rich regions in the measles virus genome [126]. However, while U-rich segments of NS1 of the influenza virus were predicted to bind RIG-I, they were not preferentially associated with RIG-I in some studies [122]. RIG-I has additional ligands in common with TLR3, apart from poly(I:C), in that RIG-I is also activated by the same PRRSV pseudoknot structure reported to activate TLR3 [83] (Figure 1C). In addition to viral RNA, RIG-I samples cellular RNAs, including those with different caps and modifications. Interestingly, it was recently shown that cellular lncRNAs can be induced to negatively regulate RIG-I signaling via binding to GA-rich motifs [134,135]. Collectively, these studies highlight that there is more to learn about recognition of both TLR3 and RIG-I, including ligands that are shared versus those that are distinct.

Like RIG-I, MDA5 (IFIH1) has a central helicase domain and two CARDs that promote signaling. Unlike RIG-I, MDA5 is preferentially activated by long (>1000 bp) regions of dsRNA [136,137]. This crucial difference seems to be mediated by a change in the angle of the CTD compared to RIG-I, such that MDA5 wraps around dsRNA and can stack along the RNA to form long filaments [138]. It is commonly implicated in sensing the genomes of positive-sense RNA viruses, such as the picornavirus family [139]. However, MDA5 may also have additional natural targets. There is evidence to suggest that MDA5 is important for the immune response to influenza A [140] and a paramyxovirus mRNA [141] (and is also a common target for paramyxovirus protein V [142]), despite both examples being negative-sense RNA viruses that do not tend to accumulate dsRNA to a high degree [31]. MDA5 is also partially involved in sensing DNA viruses, such as hepatitis B virus [143] and herpes simplex virus [144].

LGP2 is the last member of the RLR family. As opposed to RIG-I and MDA5, LGP2 does not have CARDs and is therefore signaling deficient, leaving its function not immediately clear. It has been reported to both enhance [145] and compete with [146,147] the activity of RIG-I and MDA5. Evidence seems to indicate that LGP2 stalls MDA5 movement along dsRNA, promoting filament formation and thereby enhancing signaling [148,149]. LGP2 binds to the termini of dsRNA and ssRNA [150,151] but does not require a 5′ triphosphate and does not seem to require dsRNA binding in order to inhibit RIG-I signaling [151]. Both LGP2 and MDA5 are more enigmatic in their binding characteristics and natural ligands than RIG-I [152] and could benefit from additional study.

## 4. The Importance and Variability of RNA Structure

RNA structure is highly important to RNA viruses, with even subtle perturbations capable of disrupting viral fitness or driving viral evolution [5]. At a biomechanical level, the sequence composition of dsRNA helices directly impacts mechanical properties [153,154], NMR chemical shift profiles [155], and tertiary folding [156], which contributes to these disruptions. The importance of RNA structure is further supported by therapeutic targeting of RNA structural elements for antiviral drug development [157]. Several viral replication processes rely upon the dynamic and context-specific nature of RNA structure. RNA polymerase activity is often dependent on RNA structure, with structural elements such as hairpins or pseudoknots often causing slippage, pauses, or termination [158]. RNA structure is an important element in recruiting RNA-binding proteins, facilitating genome packaging during viral assembly, translation, localization, or decay. However, the impact of RNA sequence changes that alter RNA structure on the sensing of RNA by the immune system is poorly understood.

For example, both TLR3 and RIG-I have been demonstrated to play a role in the innate immune response to influenza virus [78,159,160,161,162,163,164,165,166]. These responses are known to vary substantially across strains and levels of pathogenicity. While many factors contribute to the interconversion of low pathogenicity avian influenza viruses (AIVs) and high pathogenicity AIVs, these viruses are characterized according to mutations in the hemagglutinin (HA) gene that incorporate multiple basic amino acids into the cleavage site (CS) of precursor HA [167]. Incorporations can vary in length and composition. The mechanism of incorporation is not fully understood [167,168,169,170,171,172], though the stem-loop portion of the HA region RNA is subject to duplication and recombination [170,171,172]. Similarly, mutations associated with pathogenesis have been found in the coding regions of other AIV proteins; their role is also unclear [167,173]. Changes in nucleotide sequence associated with these mutations, whether they lead to non-synonymous or synonymous mutations, can drastically alter local and long-range interactions that contribute to RNA structure [174], changing the size and distribution of loops, bulges, and base-paired regions. Studies have reported complete or partial structure conservation across influenza strains, as well as structure variation [14,169,171,172,175,176,177,178]. It is unknown whether these RNA structural changes contribute to pathogenicity via impact on RNA sensing by TLR3 and RIG-I, but it is possible that they at least contribute to the clear difference in severity of disease across influenza strains. Further, it is possible, and likely, that similar variations in RNA structure impact RNA recognition across other infections and pathogenic disease conditions.

Also critical to RNA structure are the hundreds of natural RNA modifications, which are often hijacked by viruses. Several resources, such as the MODOMICS database [26], comprehensively track the biochemical properties, necessary enzymes, and other impacts of the hundreds of natural RNA modifications, including common examples such as N-6-methyl-adenosine, pseudouridine, and inosine. Here, we will briefly discuss the latter. Inosine is the product of deaminated adenosine, generally as a function of the protein ADAR1 acting on either foreign or endogenous dsRNA duplexes, including tRNAs [179]. ADAR1 is ubiquitously expressed and considered a major factor in immune silencing of endogenous dsRNA and a critical regulator of dsRNA-mediated pathology and immune responses [180]. It does so by binding to dsRNA via its two dsRNA-binding domains (dsRBDs) and catalytically deaminating adenosine residues to inosines (A-to-I editing). This activity seems to largely suppress RLR signaling, as deletion of MAVS rescues the embryonic lethality of ADAR1 deletion [181]. Inosine is most similar to guanosine and is read as such by replication machinery, though it base pairs less stably with cytosine. In a study of multiple common base modifications by Chiu et al., adenosine to inosine had the largest relative decrease in base hydrophobicity, indicating it would be the most destabilizing to an RNA duplex [182]. Importantly, ADAR1 seems to recognize and have preferences for certain base-pair and structural contexts beyond perfect dsRNA duplexes. The adenosine subject to editing does not need to be present in a dsRNA region, and there seems to be some preferential selection of adenosines in or near regions containing bulges and mismatches [183,184]. The context of the base 5′ to the adenosine being edited also matters, with ADAR1 preferring for this base to be A or U, followed by C then G, and particularly for As across from C mismatches [185]. ADAR1 editing is linked to an increase in replication for multiple viruses, including HIV-1 and respiratory syncytial virus [186]. These observations indicate another important link between RNA structure and immune-relevant modifications.

RNA is also a common driver of biomolecular condensate formation [187], a class of (usually liquid-liquid) phase separation caused by an increased local concentration of certain biomolecules generating a dense phase. RNA and RNA-binding proteins are commonly identified in biomolecular condensates. For example, stress-induced biomolecular condensates are strongly tied to RNA–RNA interactions, driven by RNA-binding partners and the multi-valency inherent in non-sequence-specific RNA binding [188,189]. Viruses, particularly negative-sense RNA genome viruses, are often responsible for supporting the formation of biomolecular condensation, using it to coordinate virion production to a single locale and hide from the immune system [190,191]. In some infections, the host will also form antiviral stress granules [192]. Host defenses such as RIG-I [193,194], PKR [193,195], ADAR1 [196], and TRIM25 [197] (an RNA-binding sensor in its own right [198]) are often recruited to these stress granules. As RNA–RNA interactions seem to impact the formation and function of these condensates, it is likely that RNA structure plays a significant role in the regulation of virus-induced condensates. Thus, it will be interesting to explore the impact these distinct micro-environments have on RNA structure and sensing as we learn more about their role in immune sensing [199,200].

## 5. Conclusions

The complex three-dimensional structures assumed by RNAs dictate their specific functions and molecular interactions. The formation and regulation of RNA structures are governed by a variety of physical, chemical, and structural principles, which together determine not only the specificity and affinity of interactions (RNA–RNA and RNA–protein) but also their dynamic behavior in the cellular environment. Accordingly, alterations in RNA structure can significantly impact RNA function. While powerful methods have been developed to study RNA–protein interactions and cellular RNA secondary structures, less is known about their interplay, including in the context of RNA recognition by RNA-sensing PRRs, as discussed herein. Consequently, elucidating the role of RNA structure in innate immune sensing is a critical frontier.

A historical technical challenge to this effort has been mapping RNA structure; most existing methods are low-throughput and scale in difficulty with the size and complexity of the RNA. However, great advances are being made in this regard, particularly computationally. Community efforts such as the RNA-Puzzles collaboration [201,202], a multigroup effort to improve blindly predicting RNA structure, are also making progress in de novo RNA structure prediction. Large predictive models, such as AlphaFold 3 [203], are just now starting to be able to handle sequence-to-structure modeling, but these still fail to benchmark well against user-guided predictions [204]. Novel tools are being developed, like MutaRNA, which allows for the visualization of mutation-based changes to RNA structures [174], and techniques like Detection of RNA folding Ensembles using Expectation-Maximization (DREEM), which analyzes alternate conformations of a single RNA [29]. Databases such as CircTarget [205] and MODOMICS [26] comprehensively keep track of alternative RNA compositions and their biological contexts.

Evaluating RNA-sensor function through the identification and characterization of naturally occurring ligands has thus far been a critical gap in our knowledge of these proteins. These tools and initiatives, in conjunction with future experimental forays into viral RNA structure, promise to advance our fundamental understanding of immune surveillance and host–virus interactions and facilitate the engineering of novel therapeutics for a broad spectrum of diseases. Future works on RNA/structure interactions should be able to utilize computational predictions to enhance their hypotheses and consider and incorporate the impacts of natural RNA modifications and common viral RNA structural motifs into predictive and experimental characterization of these interactions.

## Data Availability

No new data were created or analyzed in this study.
